# In silico target prediction: identification of on- and off-targets for crop protection agents

**DOI:** 10.1186/1758-2946-5-S1-P18

**Published:** 2013-03-22

**Authors:** Rucha K Chiddarwar, Andreas Bender, Sebastian Rohrer

**Affiliations:** 1 Unilever Centre for Molecular Informatics, Department of Chemistry, Lensfield Road, CB2 1 EW, Cambridge, UK; 2 BASF SE, GVM/C - A030, 67056 Ludwigshafen, Germany

## 

There has been a dramatic change in the agricultural research along the years and it has broadened its scope and scale which has become more multidisciplinary, inclusive and integrative [1]. On one hand we have literature that has shown agricultural research to have addressed key issues for society such as sustainability of production, nutrition and health [1], and on the other hand the field has generated a large amount of life science data we can now use for data mining purposes. Of relatively recent relevance for the field of agrochemicals is the area of cheminformatics where novel applications range from lead identification to target prediction [2]. Agrochemicals, just as other bioactive compounds, in many cases cause effects in biological organisms by modulating target proteins. These effects might be wanted or unwanted, depending on the target organism (pathogen or human), and hence identification of these targets is crucial for either the mode-of-action analysis or the anticipation of adverse effects of agrochemical products. In this spirit, the current project aims at a faster elucidation of agrochemical target proteins based on chemical structures. Using publicly available data as an input to chemogenomics-based in-silico target prediction models, hypotheses about putative targets for crop protection agents can be generated to guide subsequent biochemical validation experiments as well as to guide compound modifications.
